# Contrasting phenological shifts in diurnal and nocturnal Lepidoptera under climate change

**DOI:** 10.1038/s42003-026-10062-w

**Published:** 2026-04-16

**Authors:** Anders Forsman, Bafraw Karimi, Markus Franzén

**Affiliations:** 1 Center for Ecology and Evolution in Microbial Model Systems, EEMiS, Department of Biology and Environmental Science, Linnaeus University, Kalmar, Sweden; 2 Linköping University, IFM Biology, Linköping, Sweden

**Keywords:** Phenology, Community ecology, Behavioural ecology, Biodiversity, Evolutionary ecology

## Abstract

Phenological shifts enable species to adjust the timing of life-history events to required resources. Climate change alters the spatiotemporal association between temperature and photoperiod, modifying the scope for temperature regulation. Here, we propose a model hypothesizing that dissimilar light requirements and constraints differently impact phenological responses in diurnal versus nocturnal ectotherms. Next, we investigate temporal shifts and latitudinal trends in phenology for 363 Lepidoptera species using 44 years of citizen science data. In agreement with model predictions, long-term shifts in the estimated onset, peak, termination, and duration of the flight period differed qualitatively between diurnal and nocturnal species, even after accounting for voltinism and overwintering stage, supporting that diel activity is a key regulator of phenology. Phenology showed intraspecific latitudinal trends, and the peak occurred later in the north in diurnal species but was independent of latitude in nocturnal species. These contrasting phenological shifts may impact community composition and ecosystem functioning.

## Abstract (web summary)

A novel model of how light requirement, temperature, and photoperiod jointly define phenological time-spaces for diurnal and nocturnal ectotherms, combined with long-term data for Lepidopterans demonstrates how diel activity modulates phenological responses to climate change.

## Introduction

When faced with thermal changes, species can advance, delay, prolong, or shorten their activity to match their optimal temperature range^1–4^. Phenology varies considerably among species depending on taxonomic group, and different components of climate drive phenology in different regions^5^. For example, species at higher latitudes respond most strongly to temperature, and the phenology of ectotherms is more influenced by temperature compared with endotherms^5^. A trend towards phenological advancement has been observed among, for example, plants^6,7^, birds^7,8^ and insects^7,9,10^ in the past decades. As photoperiod and temperature are common cues to phenology, phenology is expected to show strong latitudinal patterns^5,11–13^. Latitude can also define the phenological responses to climate change, particularly in ectotherms, as they rely on ambient temperatures, microhabitats, and sun basking for body temperature regulation^5,12,14–16^. Because temperature increases at a faster rate towards the poles compared to equatorial locations, it is pivotal to investigate how species at high latitudes are affected by temperature change^17–19^. Forsman et al.^20^ report that rates of poleward range shifts in terrestrial animals differ between taxonomic groups, vary according to species traits associated with ecological generalization, increase over time, and depend on latitude. Similar context dependence applies to phenology shifts^5^.

Butterflies and moths are well studied, wide-spread, vary in ecology, life-history and behaviours, comprise both diurnal, crepuscular and nocturnal species^12,21^, are frequently monitored and reported in citizen science databases^2,22^, and display a diversity of phenological responses^3,21,23–36^. Although there are some inconsistencies among studies, Lepidopteran phenology shifts correlate with latitude, diapause, overwintering stage, migration, host plant use, dietary breadth, body size, seasonal appearance (early or late), and number of generations (voltinism)^12,18,21,25,29,30,36–41^. However, whether and how diurnal/nocturnal behaviour modulates phenological shifts in time and space has not been systematically investigated. Yet, climate change alters the spatial relationship between temperature and photoperiod, induces cue-environment mismatches, and modifies the opportunity and need for temperature regulation. Global warming also shows a diurnal asymmetry, with night-time temperatures having increased more rapidly than daytime temperatures^42^. How global warming impacts organisms may thus be modulated by time-specific behaviours and activity patterns^43^. We therefore hypothesize that climate change will differently impact phenological responses in diurnal and nocturnal ectotherms.

Research on phenology and climate change escalated from fewer than 50 papers per year in 1995 to over 1600 papers published in 2025 (Fig. S1), underscoring the growing recognition of this topic. Only a minority (2.5%, 29 of 1140) of the studies of Lepidoptera (butterflies or moths) investigated aspects related to nocturnal behaviour (Fig. S1). We are aware of three studies that compare phenology between diurnal and nocturnal Lepidoptera, and, unlike this contribution, none of these earlier studies report any significant difference^28,30,40^.

Here, we propose a conceptual model of how light requirements and photoperiod modulate phenological responses to variation in temperature associated with climate change and latitude. Our model builds on the hypothesis that joint effects of temperature and photoperiod define different phenological time-spaces for diurnal and nocturnal ectotherms. To test model predictions, we use citizen science records on spatiotemporal variation in phenology covering over four decades (1981–2024) and 363 species of butterflies and moths in Sweden, in northern Europe with a highly seasonal climate.

## Results

### Modelling how diel activity modulates phenological responses to climate change

**Fig. 1:**
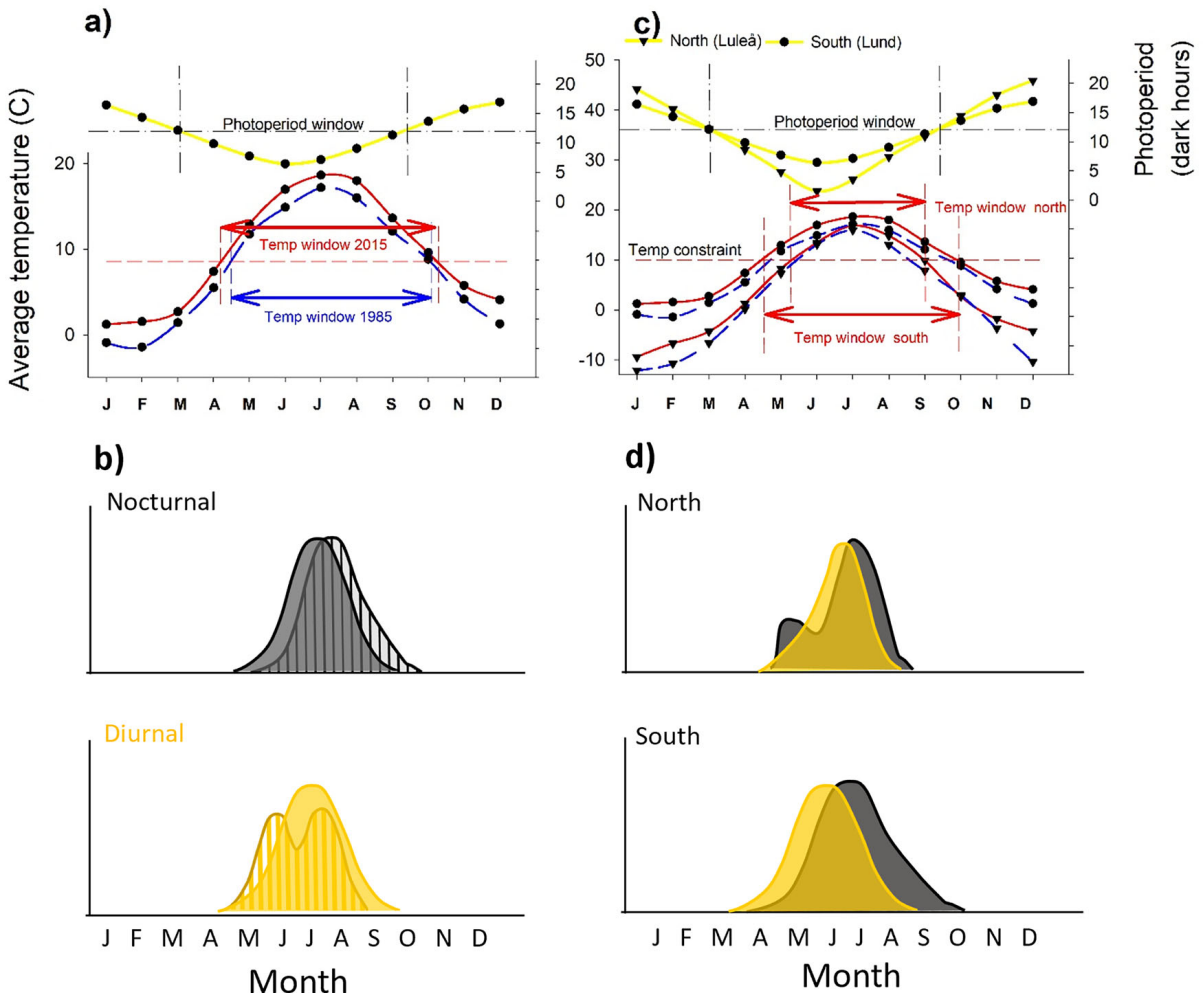
Conceptual model showing how variation in temperature and photoperiod jointly defines different phenological time-spaces for diurnal and nocturnal ectotherms. **a** The seasonality of ambient air temperature and photoperiod is asynchronous, with temperature peaking later than photoperiod. Shifts in the timing of activity in response to climate change that broaden the temperature window of opportunity (indicated by the red and blue bidirectional arrows and defined by the species’ thermal requirements indicated by the red hatched horizontal line) may therefore be modulated by photoperiod. Seasonality of photoperiod and temperature change is based on data for Lund (southern Sweden, latitude 55.7°N) in 1985 and 2015. **b** Phenological responses to rising temperatures may be modulated by light requirements and photoperiod. We hypothesize that it is more difficult for diurnal species (mainly butterflies), which are constrained by short days, to initiate activity earlier in the spring and to delay the termination of the activity period until later in the autumn than for nocturnal species (most moths). Diurnal species should shift the peak of their flight period to earlier in the season, thereby better matching the peak of the photoperiod, and increased activity due to relaxed temperature constraints should shorten their flight period. Rising temperatures reduce the time needed to complete the life cycle, and a prolonged temperature window of opportunity may broaden conditions for multi-voltinism and generate bi- or trimodal phenology distributions ^30^, particularly in diurnal species. Barred areas indicate temporal modification of phenology induced by climate change relative to the original (filled areas). The abundance y-axis represents the number of individuals of a hypothetical species. **c** The seasonality of ambient temperature and photoperiod at southern and northern latitudes is synchronous but differs in magnitude and by about one month in timing. The temperature window of opportunity (indicated by the red bidirectional arrows, defined by the species’ thermal requirement) is narrower in the north than in the south. Seasonality of photoperiod and temperature based on data for Lund (55.7°N) and Luleå (65.6°N) in 1985 and 2015. Note that the scale of the temperature y-axis is different in a) and c). **d** Due to the latitudinal gradient in day length and the displacement of the seasonal peaks in temperature and photoperiod, latitude should impose stronger constraints on phenology in diurnal compared with nocturnal species, especially concerning the peak and duration of the activity period. Assuming long days limit nocturnal species, the phenology of nocturnal species should be bimodal at northern latitudes, with high activity in spring and autumn. Within the spatiotemporal scale considered here, the difference in the length of the temperature window of opportunity attributable to latitude (from southern to northern Sweden) is larger than the difference instigated by climate change between 1985 and 2015 (red and blue curves). Long-term large-scale phenology data for Lepidopterans enable evaluations of these model predictions and inferences on how climate change may impact activity periods of nocturnal and diurnal species at different latitudes.

Although Lepidopteran phenology is defined by a complex interplay of multiple species traits, environmental variables, and interspecific interactions affecting different life-stages, an all-inclusive model is beyond the scope of this contribution. The focus here is on how diel activity may impact on temporal and latitudinal phenology shifts of the adult flight period (Fig. 1).

In our study area, the seasonality of average ambient air temperature and photoperiod are asynchronous, with temperature peaking about one month later than photoperiod (Fig. 1a). Shifts in the timing of the flight activity period in response to climate change that broaden the temperature window of opportunity over time may, therefore, be modulated by the seasonal variation in photoperiod and by the species’ light requirements. Depending on how many light or dark hours a species requires per day, the potential to advance the onset or delay the termination of the activity in response to rising temperatures may not be fully realized due to constraints by the window of opportunity defined by the seasonality of photoperiod (Fig. 1b). Furthermore, depending on the thermal preferences and tolerances of the species, rising temperatures may facilitate or limit adult activity^32^, thereby potentially affecting the onset, peak, termination, and duration of the flight period. This should differently impact diurnal thermophilic organisms that can regulate their body temperature by sun-basking and nocturnal thermo-conformers whose body temperature depends more on the air and substrate temperature^16,21^. For example, rising temperatures should allow diurnal species to shift the peak of their flight period to earlier in the season, thereby better matching the peak of the photoperiod. Increased activity due to relaxed temperature constraints should also shorten the duration of the flight period, particularly in diurnal species. Diapause and overwintering life-stage is important for Lepidopteran phenology^29,39,40^, and shifts in the onset of the flight period may be more related to the rate of larval development than to diurnality, compared with the termination. We recognize that some nocturnal species might bask while inactive during the day, and that photoperiod could constrain nocturnal species by determining night length as much as day length. We nevertheless hypothesize that, in general, it is more difficult for diurnal species (mainly butterflies) that are potentially constrained by short days to shift their onset of activity earlier in the season and to delay the termination of their activity until later in the autumn, compared with nocturnal species (most moths). Furthermore, rising temperatures will speed up physiological processes and shorten the time needed to complete the life cycle^11,44^. Combined with a prolonged temperature window of opportunity due to climate change, this may broaden the conditions for multi-voltinism^9,26,30^, particularly in diurnal species exposed to higher temperatures (Fig. 1b).

### Modelling impacts of diel activity on latitudinal phenology trends

Our model predicts that latitude will differently influence the phenology of nocturnal and diurnal organisms. In our study area, the seasonality of average ambient air temperature and photoperiod at southern and northern latitudes is synchronous but differs in magnitude (Fig. 1c). Therefore, the temperature window of opportunity is shorter in the north. This should impact all four phenology dimensions and particularly reduce the duration of the flight period. Under the assumption that short days do not restrict the adult activity of nocturnal species, they should be less sensitive to the seasonality of photoperiod compared with diurnal species that rely on sun-basking for temperature regulation. Also, because of the latitudinal gradient in day length and the temporal displacement of the seasonal peaks in temperature and photoperiod, latitude should impose a stronger constraint on intraspecific phenology trends in diurnal compared with nocturnal species, especially with regard to the peak and duration of the flight period (Fig. 1d).

### Model evaluation

To evaluate the model, data for Lepidopterans were used to test the following hypotheses and predictions: (i) Phenology has changed over time, and the temporal shifts are different in diurnal and nocturnal species; (ii) Shifts in the onset of the flight activity period are independent of shifts in the termination of the activity period; and (iii) Intraspecific latitudinal gradients in contemporary phenology differ between diurnal and nocturnal species.

**Fig. 2:**
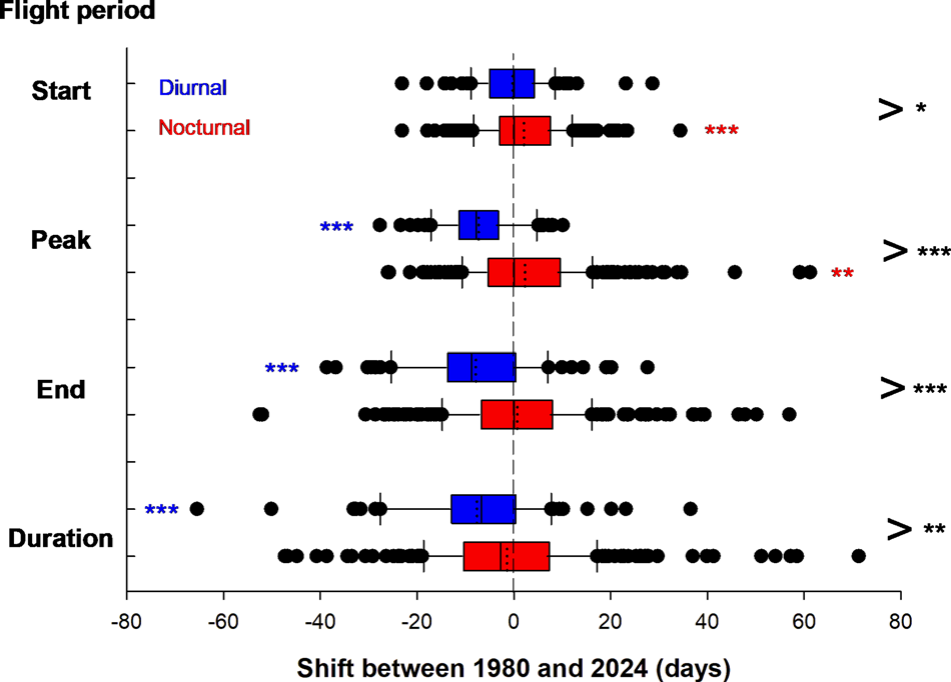
Comparisons of phenological shifts in the adult flight period of diurnal (*n* = 80) and nocturnal (*n* = 283) Lepidopterans between 1981 and 2024. The onset (start), peak, and termination (end) were estimated using quantile regressions as the predicted 0.05, 0.5 and 0.95 percentiles, in ordinal days, in 1981 and 2024. The duration was estimated by subtracting the onset from the termination of the flight period. The dashed vertical line (at shift = 0) indicates no change in phenology. Negative and positive values indicate advances and delays of phenology, in days. Plots show centre line, median; dotted line, mean; box limits, upper and lower quartiles; whiskers, 1.5x interquartile range; points, outliers. Asterisks in red and blue indicate results from paired comparisons *t*-tests of intraspecific shifts (see Table S2). Asterisks in black to the right indicate results from comparisons of shifts between diurnal and nocturnal species after taking into consideration the effects of voltinism and overwintering life stage using MIXED (see the effects of the interaction between diurnality and year in Table 1). *p* < 0.001 (***), *p* < 0.01 (**), *p* < 0.05 (*).

Phenological patterns were analysed based on data for diurnal (*n* = 80 species, 69 butterflies and 11 diurnal macro moths, consisting of 941,107 records) and nocturnal (*n* = 283 species, only macro moths, consisting of 844,078 records) Lepidoptera from Sweden covering the period 1981 to 2024. Quantile regression analysis^45^ was applied to model and predict four phenology metrics (onset (first), termination (last), peak, and duration of the flight period, corresponding to the 0.05, 0.5, and 0.95 quantiles, and the difference between the 0.95 and the 0.05 quantiles) for each species at in two separate years (1981 and 2024) (for details see **Methods**). Analyses of the estimates showed that taxonomic affinity (family) accounted for, on average 19.5% and 8% of the total phenological variance in diurnal and nocturnal Lepidoptera (Table S1, Fig. S2). The corresponding estimates for long term phenology shifts were 16% and 4.8% (Table S1). Between 1981 and 2024, most (240 out of 363 (66%) of the investigated species shifted to a later spring emergence by on average 6 days (maximum 34 days) (*t*-test, *P* < 0.05), and most (220) species (61%) shifted to an earlier termination of the flight period by on average 10 days (maximum 53 days) (*t*-test, *P* < 0.0001; Fig. S2). As a consequence, 238 species (66%) shortened their flight duration by on average 11 days (maximum 65 days) (*t*-test, *P* < 0.01; Table S2, Fig. 2). Additionally, most (224) species (62%) shifted the peak of the flight period to an earlier date (*P* < 0.0001), by on average 7 days (maximum 28 days).

For visual representations of the distributions of the four phenology variables in 1981 and 2024 for diurnal and nocturnal species, see Fig. S3. Correlation analyses indicated significant associations between all four phenology variables in both diurnal and nocturnal species (all *P* < 0.05), except between peak appearance and flight duration in nocturnal species (*P* = 0.91; Table S3). However, shifts in the onset from the beginning (1981) to the end (2024) of the study period were independent of shifts in the termination of the flight period (Diurnal: *r* = -0.063, *P* = 0.58, *n* = 80; Nocturnal: *r* = 0.07, *P* = 0.24, *n* = 283, Fig. S4).

### Shifts in phenology according to diel activity, voltinism, and overwintering life stage

The estimated long-term shifts of the flight period differed significantly between diurnal and nocturnal species both in magnitude (onset, termination, duration) and direction (peak of the flight period) (effect of the year x diurnality interaction (all four *P* < 0.05), Table 1, Table S2, Table S4, Fig. 2).

**Table 1:** Variation in phenology in lepidopterans according to year, diel activity, voltinism, and overwintering stage

	Effect	*F*	*df*	Estimate	s.e.	*η*^2^	*P*
**Onset**	Year	3.17	361	2.10	0.49	0.0001	0.0756
	Diel activity (DA)	1.75	18.1	-7.26	5.493	0.0057	0.2026
	Voltinism	13.64	356	14.63	3.959	0.0422	**0.0003**
	Overwinter as larva	15.71	325	15.11	3.812	0.0482	**<0.0001**
	Year ˟ DA	4.91	361	-2.33	1.052	0.0002	**0.0273**
**Termination**	Year	16.58	361	0.72	0.827	0.0018	**<0.0001**
	Diel activity (DA)	0.02	16.7	-0.69	5.287	0.0001	0.8978
	Voltinism	11.40	355	13.56	4.018	0.0267	**0.0008**
	Overwinter as larva	7.53	317	10.58	3.856	0.0226	**0.0064**
	Year ˟ DA	23.90	361	-8.61	1.762	0.0026	**<0.0001**
**Peak**	Year	12.24	361	2.31	0.669	0.0009	**0.0005**
	Diel activity (DA)	0.15	17.9	-1.97	4.99	0.0004	0.6985
	Voltinism	0.00	355	0.11	3.944	0.0000	0.9778
	Overwinter as larva	12.01	318	13.09	3.778	0.0344	**0.0006**
	Year ˟ DA	45.35	361	-9.60	1.426	0.0034	**<0.0001**
**Duration**	Year	20.26	361	-1.38	0.944	0.0102	**<0.0001**
	Diel activity (DA)	9.99	503	7.64	2.418	0.0140	**0.0017**
	Voltinism	212.24	359	28.65	1.967	0.3255	**<0.0001**
	Overwinter as larva	6.94	359	-4.93	1.871	0.0155	**0.0088**
	Year ˟ DA	9.76	361	-6.28	2.010	0.0049	**0.0019**

Results from mixed model analyses of variance implemented with procedure MIXED in SAS. Estimated phenology variables include onset, termination, peak, and duration of the flight period (estimated using separate quantile regressions for each species). The predictor variables were year (1981 or 2024), diel activity (diurnal/nocturnal), voltinism (0.5, 1 or 2), and overwintering stage (larva / not larva). The table shows *F*-values, the Satterthwaite approximations for the denominator degrees of freedom (*df*), slope estimates with standard errors (s.e.), and *P*-values for each predictor. *η*^2^ represents the approximate partial Eta-square local effect size estimated using the procedure GLM in SAS^79,81^. *P*-values less than 0.05 are highlighted in bold. Most (18 of 19) of the significant associations remained statistically significant after Bonferroni corrections (critical *P* = 0.05/4 = 0.0125). The random effect of species was significant for all phenological measures (Wald test, all *P* < 0.0001). The random effect of family was non-significant in all analyses.

Results from paired *t*-tests suggested that in diurnal species, the estimated onset did not change significantly between 1981 and 2024 (*P* = 0.80). However, diurnal species exhibited statistically significant shifts towards an earlier peak (7.3 days, *P* < 0.0001), an earlier termination (7.9 days, *P* < 0.0001), and a shortened duration (7.7 days, *P* < 0.0001) of the flight period over the past four decades (Fig. 2, Table S2). In contrast, nocturnal species shifted to a significantly later onset (2.1 days, *P* < 0.0001) and a postponed peak (3.2 days, *P* = 0.0015) of the flight period, but neither the duration nor the termination of the flight period of nocturnal species changed significantly (*P* > 0.05) between 1981 and 2024 (Fig. 2, Table S2).

With regards to life history traits, results from mixed model Anova’s showed that, in addition to the effects of diurnality, both phenology and the long-term phenological shifts depended on voltinism (all *P* < 0.001, except for peak phenology *P* = 0.98) and overwintering life stage (larva vs. not larva, all *P* < 0.01) (Table 1, Table S4). Most notably, multi-voltinism was associated with pronounced temporal shifts towards a later peak (*P* < 0.0001), a postponed termination (*P* < 0.0001), and a prolonged duration (*P* < 0.0001) of the flight period, whereas semi- and univoltine species shifted in the opposite direction (Table S4). Species that did not overwinter as larva showed a more delayed onset (*P* < 0.0001) and a more shortened duration (*P* = 0.0003) of the flight period between 1981 and 2024, compared with species overwintering as larva (Table S4).

The modulating effects of diel activity on phenology and phenological shifts in onset, peak, termination, and duration of the flight period over the last four decades reported above remained qualitatively unchanged when the associations with voltinism and overwintering life stage were accounted for in the statistical models (Table 1, Table S4).

### Intraspecific latitudinal variation in phenology

**Fig. 3:**
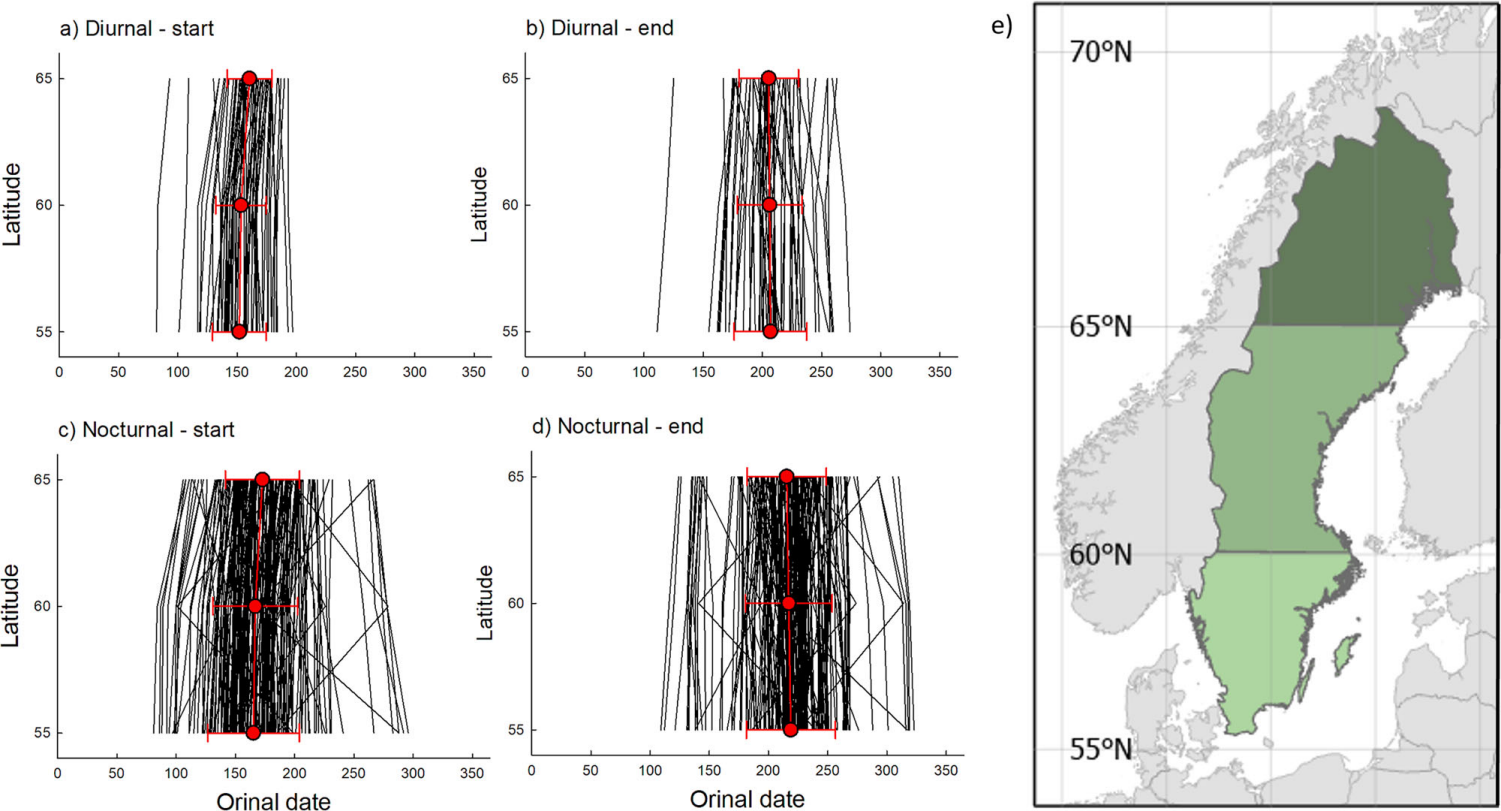
Latitudinal intraspecific variation in the adult flight phenology of diurnal and nocturnal Lepidopterans. Figure shows how the onset (start, **a**, **c**) and the termination (end, **b**, **d**) of the adult flight period varies with latitude for 54 diurnal species (**a**, **b**) and 176 nocturnal species (**c**, **d**) in 2024. The onset and termination represent the predicted 0.05 and 0.95 percentiles in 2024 obtained using separate quantile regressions for each species. Each thin black line connects data for one species across three latitudinal bands (centred at 55, 60, and 65°N (**e**)). Red circles represent mean values (±s.d.) across species for each latitude. The significant effects of latitude and diel activity are reported in Tables 2 and S5.

**Fig. 4:**
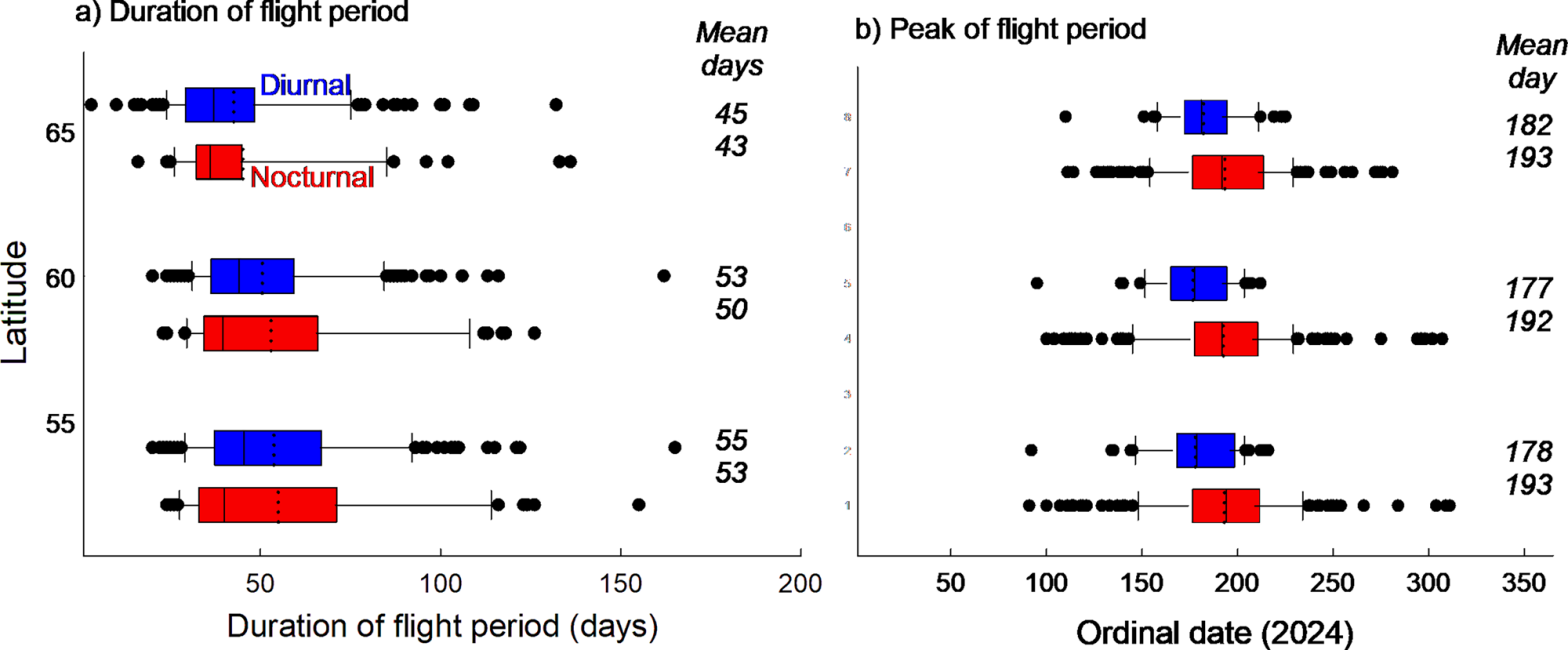
Latitudinal variation in contemporary duration and peak of the adult flight activity period in diurnal and nocturnal Lepidopterans. Box plots show how the duration (**a**) and the peak (**b**) of the adult flight period varies with latitude for 54 diurnal and 176 nocturnal species across three latitudinal bands (centred at 55, 60, and 65°N) in 2024. The duration represents the difference between the 0.95 and the 0.05 percentiles, and the peak represents the 0.5 percentile. Plots show centre line, median; dotted line, mean; box limits, upper and lower quartiles; whiskers, 1.5x interquartile range; points, outliers. Values in italics indicate the average duration of the flight period (**a**) and the average peak (ordinal date) of the flight period (**b**). The duration of the flight period varied significantly according to latitude, and the peak of the flight period varied significantly according to latitude, diel activity, and their interaction (see Tables 2, S5).

To evaluate and compare contemporary intraspecific latitudinal trends in phenology, data for a subset of diurnal (*n* = 54 species, 469,355 observations) and nocturnal (*n* = 176 species, 527,300 observations) Lepidopterans were used to model separate quantile phenology metrics for each of three 5 ^O^ (≈555 km) wide latitudinal bands (centred at 55, 60 and 65°N) in 2024. All four phenology dimensions changed significantly with latitude (mixed model Anova’s, all *P* < 0.05), and all except the duration of the flight period differed significantly between diurnal and nocturnal species (onset (*P* < 0.0001) and termination (*P* < 0.001) Fig. 3, duration (*P* = 0.54) and peak (*P* < 0.001; Fig. 4, Tables 2, S5).

**Table 2:** Associations of contemporary phenology (onset, termination, peak, and duration of the flight period) with latitude and diel activity (diurnal or nocturnal)

*Phenology variable* Effect	*F*	*P*	Estimate ± s.e.	LS-means (diurnal vs nocturnal)
*Onset*				
Latitude	*F*_1456_ = 139.82	0.0001	0.77 ± 0.066	
Diel activity	*F*_1582_ = 5.65	0.0178	−21.84 ± 9.19	152 vs 169
Lat*Diel activity	*F*_1456_ = 0.34	0.5578	0.08 ± 0.14	
*Termination*				
Latitude	*F*_1455_ = 8.07	0.0047	−0.36 ± 0.084	
Diel activity	*F*_1567_ = 6.87	0.0090	−30.00 ± 11.450	202 vs 219
Lat*Diel activity	*F*_1455_ = 1.57	0.2105	0.22 ± 0.175	
*Peak*				
Latitude	*F*_1456_ = 6.29	0.0125	0.02 ± 0.086	
Diel activity	*F*_1562_ = 12.71	0.0004	−41.24 ± 11.565	177 vs 193
Lat* Diel activity	*F*_1456_ = 5.43	0.0202	0.42 ± 0.178	
*Duration*				
Latitude	*F*_1457_ = 119.22	0.0001	−1.13 ± 0.094	
Diel activity	*F*_1522_ = 0.38	0.5365	−7.49 ± 12.113	50 vs 49
Lat* Diel activity	*F*_1457_ = 0.51	0.4753	0.14 ± 0.194	

Results from PROC MIXED in SAS based on data for 54 species of diurnal (mainly butterflies) and 176 species of nocturnal (moths only), all of which contributed with phenology estimates for 2024 for three latitudinal bands (55, 60 and 65°N). Phenology attributes include onset, termination, peak, and duration of the flight period. LS-means indicate least-squares means. The random effect of species was significant for all phenological measures (all *P* < 0.0001). Results from more complex models, including voltinism and overwintering stage as additional explanatory variables, are reported in Supplementary Table S5.

On average, diurnal species both started and terminated their flight period about 17 days earlier compared with nocturnal species (mixed model Anova, both *P* < 0.05), but there was considerable variation among species within each group, and the latitudinal onset and termination trends did not differ significantly between diurnal and nocturnal species (diurnality x latitude interaction, both *P* > 0.05; Fig. 3, Table 2). The duration of the flight period was about 11 days shorter on average at the most northern latitudinal band (average duration ca 44 days) compared with the intermediate and southernmost latitudinal bands (ca 53 days on average), in both diurnal and nocturnal species (*P* < 0.0001; Fig. 4a, Table 2). The flight period peaked 16 days later in nocturnal compared to diurnal species on average, and their latitudinal trends were different (significant latitude x diurnality interaction, *P* < 0.05; Table 2), with the peak being independent of latitude in nocturnal species (average ordinal date = 193) while occurring ca 5 days later at the most northern latitudinal band in diurnal species (Fig. 4b). The difference in the peak of the flight period between diurnal and nocturnal species was twice as large at the most northern band (11 days) compared with the intermediate and southern bands (5 days) (Fig. 4b, Table 2). Results from mixed model Anova’s showed that the different intraspecific associations of phenology with latitude in diurnal and nocturnal species reported above remained qualitatively unchanged when the potentially modulating effects of overwintering life stage and voltinism were statistically accounted for (Table S5).

## Discussion

Previous work has shown that phenological shifts of Lepidoptera due to climate change are modified by environmental variables and a suite of species traits^12,18,21,25,29,30,36,38,39,46,47^, but the role of nocturnal behaviour has not been systematically evaluated. Here we present a conceptual model showing that the joint effects of light requirement, temperature and photoperiod define different phenological time-spaces for diurnal and nocturnal ectotherms. Further, our analyses of citizen science records demonstrate that the phenology of the adult activity period of Lepidoptera species in Sweden has changed over the past four decades, and differently so in diurnal and nocturnal species, as predicted by our model. Although there was considerable variation in the estimated long-term trends among species, the majority (66%) shifted to a later onset (on average 6 days), most (62%) species shifted to an earlier peak (on average 7 days), and most species (61%) terminated their flight period earlier (on average 10 days), resulting in a shortened duration of the flight period (on average 11 days) in the majority (66%) of species. The results also showed strong overall latitudinal intraspecific trends in all investigated phenology metrics, in agreement with model predictions. Phenology shift magnitudes appear smaller with quantile-based estimation methods compared to single-time point comparisons (like first or last observations), complicating comparisons across studies. Despite this, our results are qualitatively largely consistent with previous studies^5,7,8,13,21,28^, underscoring the impact of climate change on phenological shifts.

As hypothesized, the long-term phenological shifts differed in both magnitude and direction according to diel activity, and were generally more pronounced in diurnal (peak, termination, duration) than in nocturnal (onset) species. The difference in intraspecific latitudinal trends, with the peak of the activity period being independent of latitude in nocturnal species and shifting to a later date at more northern latitudes in diurnal species, further supports that photoperiod and light requirements modulate the phenological response to changing temperatures in diurnal and nocturnal species. Together, the comparisons of temporal and latitudinal trends between diurnal and nocturnal species strengthen the conclusion that diel behaviour is a key regulator of phenology shifts in Lepidoptera.

Our combination of analytical approaches was designed to evaluate and mitigate potential biases in phenological estimates due to observer behaviour in community science data, variation in number of records, relatedness, and species traits. It is possible that our results were partly influenced by how the original observational data for butterflies and moths were collected^48^ or by associations of phenology with traits (other than nocturnal behaviour voltinism, and overwintering life stage) or environmental variables that were not considered in our analyses. However, while including additional traits may increase the total variance explained it is unlikely to remove the apparent relationship between phenological shifts and diurnality. A comprehensive phylogeny of Lepidoptera suggests that butterflies represent a monophyletic group of diurnal species, nested within a paraphyletic group of (mostly) nocturnal moths^49^, and so the differences reported in the present study may partially reflect shared ancestry. However, previous studies of phenology in Lepidopterans report very low signals of phylogenetic relatedness^28,36^. Together with the pronounced interspecific variability and strong intraspecific latitudinal trends observed in the present study, this indicates that developmental plasticity, responsiveness, and evolvability of phenology are high in Lepidoptera. Also, our main hypothesis concerns spatiotemporal *shifts* in phenology rather than phenology per se, reducing the potentially complicating issue of shared ancestry. Furthermore, an analytic approach based on phylogenetic independent contrasts is not feasible given the distribution of nocturnal/diurnal behaviour in the Lepidoptera phylogeny.

Despite the extensive literature on Lepidopteran phenology, few previous studies have systematically compared diurnal and nocturnal species. An investigation of distributional and phenological shifts in 289 Finnish Lepidoptera over 25 years reports that 73% of species did not advance their peak phenology, and that phenology shifts did not differ between butterflies and moths (see Table S2 in ref. ^28^). We investigated a broader set of phenology metrics and demonstrated differences in long-term shifts between diurnal and nocturnal Lepidopterans in all traits, including peak appearance. Our study includes a larger number of species (363), and our study area encompasses regions with different climatic conditions, such as earlier snowmelt in southern Sweden, which could contribute to more pronounced phenological shifts. It has previously been established that estimated rates of responses to climate change depend on methodology, study area, study period, and study duration^18,20,24,48,50^. Another recent investigation of variation in Lepidopteran phenometrics representing 265 species and the entire North American continent over the period 1902–2016 reports that the midpoint, termination, or duration of the adult flight period was independent of diurnality (based on 95% Bayesian credible intervals) when accounting for other species traits and environmental variables using Phylogenetic LMMs^40^. However, that study^40^ did not evaluate the interactions between diurnality and year nor between diurnality and latitude to specifically test the main hypothesis put forward in the present study that long-term and latitudinal phenological *shifts* should differ between diurnal and nocturnal species.

The results of the present study suggested that long-term shifts in the onset of the flight period were independent of shifts in the termination of the activity period, likely allowing for flexibility in the duration of the flight period. We cannot ascertain whether the phenological variation and shifts primarily reflect high inter-individual variation in temperature preferences and timing of activity^32,51,52^, or within-population synchrony combined with modifications of voltinism over time and space^2,9,26,30,53,54^. Regardless of the underlying mechanism(s), the high phenological variability and sensitivity suggest populations and species are capable of adjusting to future environmental change^24,55,56^.

The intraspecific latitudinal trends showed that, on average, the onset of the flight period was delayed, while the termination and duration were advanced and shortened, respectively, at higher latitudes. This conforms with earlier studies^5,13^, and resembles the demonstration that biogeography influences diel time partitioning strategies in mammals^57^. However, Valtonen et al.^12^ report that of 65 moth species, there was evidence for geographic variation in phenological thresholds in only 17 species (26%). Our results also indicated that the peak of the flight period was generally more constrained by latitude in diurnal compared with nocturnal species, in agreement with the model prediction. To our knowledge, there exists no previous comparison of latitudinal phenology trends between these groups.

Lepidoptera include important pollinators, food resources for arthropods, birds and bats, parasites, parasitoids, and pests in agriculture and forestry. Any effects that the contrasting long-term phenological shifts and latitudinal trends in the peak of the flight period have on community compositions may therefore impact species interactions and ecosystem services, with potentially negative consequences for socio-economy and human well-being^9,21,58–62^.

In summary, we present a hypothesis of how the joint effects of light requirement, temperature, and photoperiod define different phenological time-spaces for diurnal and nocturnal ectotherms. Our large-scale and long-term data for Lepidopterans in Sweden provide rare evidence for contrasting temporal and latitudinal phenological shifts in diurnal and nocturnal species in general agreement with model predictions, offering overall support for the hypothesis that diel activity is a key regulator of phenology responses to environmental change. Additional work is necessary to assess the validity of our hypothesis, whether the association of phenological shifts with nocturnal/diurnal behaviour is robust to the inclusion of additional traits, and how the differential responses impact community composition, trophic interactions, and ecosystem functioning. To evaluate generality, future studies may explore how diel activity modulates phenological responses to climate change in Lepidopterans at other latitudes, in other ectothermic taxa (e.g., lizards, snakes, and amphibians), and in endotherms such as birds and bats that do not rely on ambient conditions for body temperature regulation.

## Methods

### Study area, data assembly, species selection, and filtering of observations

To test model predictions and evaluate patterns and trends in adult flight period timing in diurnal and nocturnal Lepidoptera, phenological observations were assembled spanning ca 14 degrees latitude (55° to 69°) across Sweden, northern Europe, covering the period January 1981 to December 2024. For the period 1981–2020, we relied on our own curated historical dataset originally compiled via Analysportalen (https://fynddata.artdatabanken.se/analysisportal, now discontinued), which we carefully harmonized and quality-checked to correct for taxonomic inconsistencies and observational biases. For the period 2021–2024, we used records extracted from the Global Biodiversity Information Facility (GBIF, https://www.gbif.org/, 10.15468/dl.pfc2ve; extracted 7 June 2025), which by then had undergone substantial improvements in data quality and taxonomic validation. The combined dataset generated by this two-tier approach ensured reliable long-term coverage while minimizing the risk of artefacts, since earlier records and online submissions often required extensive curation, whereas post-2020 records can be considered of consistently higher quality. We focused on conspicuous macro-Lepidoptera species that are reliably recorded in Swedish monitoring programmes and citizen science initiatives.

We retained only adult/imago observations with a valid date (day of year) and coordinates (latitude and longitude). To minimize New Year boundary misdating, January and December records were excluded. Species-specific phenological outliers were removed using the 1st–99th percentiles of start and end day of year derived from the curated reference data. Species were considered eligible if they had at least ≥30 total records, ≥5 distinct observation days, and ≥5 distinct years of data. In practice, the included species exceeded these limits (the lowest values among included species were 120 records, 30 distinct observation days, and 32 years).

After applying taxonomic harmonisation and the above quality filtering, the combined dataset comprised 363 species and 1785,185 records spanning 1981–2024. This included 80 diurnal species (69 butterflies and 11 diurnal macro-moths; 941,107 records) and 283 nocturnal macro-moth species (844,078 records) representing 19 families (see Statistical Analysis and Reproducibility)^63^. Day and month of records were transformed to ordinal day of year records (from 1 to 365). To analyse and compare long term phenology shifts based on this data, four phenology metrics were estimated using linear quantile regressions to represent the onset, termination, peak, and duration of the flight period for each species at the beginning (1981) and at the end (2024) of the study period (see below).

To analyse and compare contemporary intraspecific latitudinal phenology trends, we used records from 2013 to 2024 (*n* = 996,655 observations). Observations were assigned to one of three latitudinal bands (from decimal latitude: 52.5–57.5° N (55 °N band), 57.5–62.5° N (60 °N band), and 62.5–67.5 °N (65 °N band)), each spanning 5 ° ( ~ 555 km). To ensure robust trend estimates, only species for which estimates could be predicted for each of the three latitudinal bands, and with estimates based on at least 15 records per latitudinal band across 2013–2024, were included in the downstream analysis. This yielded a data set of 54 diurnal and 176 nocturnal species^63^.

### Estimating phenometrics from day of year observational records

Estimates of phenology are potentially subject to variation in abundance, sampling strategy, sampling intensity, sample sizes, and recorder biases^41,64,65^. Despite these issues, opportunistic citizen science data allow for investigating patterns over spatial and temporal scales that would not be tractable using traditional sampling^5,7,25,40,41,65–69^.

Quantile regression analytical approaches based on estimation of percentiles of day of year observations generate more robust estimates and evaluations of trends compared with analyses of extreme values^45^. As such, they offer a means to mitigate potential biases associated with opportunistic (e.g., citizen science) data sets and have been used previously to analyse insect phenology e.g., refs. ^28,34,39,40,65,70^. To quantify interspecific variation and long-term shifts in the adult flight period, linear quantile regression analysis (implemented with the *quantreg* package in R, version 6.1) was applied separately for each species to data for the period 1981 to 2024. These regressions were used to generate predicted approximations of four phenology metrics; the onset, termination, peak, and duration of the flight period (estimated as the 0.05, 0.5, and 0.95 quantiles, and the difference between the 0.95 and the 0.05 quantiles) separately for each species at two reference years (1981 and 2024). Next, species-specific long-term phenological shifts were estimated as the difference between the predicted phenology metrics for the beginning (2024) and the end (1981) of the study period. The predictions for the two time points were also used to quantify, visualize, and compare variation in the adult flight phenology of diurnal and nocturnal species in 1981 and 2024.

To quantify intraspecific latitudinal variation in phenometrics, linear quantile regression analysis (at τ = 0.05, 0.50, 0.95, implemented with the *quantreg* package in R, version 6.1) was applied to the second data set (2013–2024). For each species at each of the three latitudinal bands (55°, 60°, and 65°N) predicted estimates were generated for the ordinal day of the flight period onset (0.05 quantile), peak (0.50 quantile), and termination (0.95 quantile) for 2024. The predicted flight period durations (in days) were then calculated as the difference between the predicted termination and onset dates.

The above estimates were then used as input data in the downstream analyses to model variation in phenology, long-term phenological shifts, and latitudinal trends according to family, diel activity, voltinism, and overwintering as larva (see Statistical analysis and Reproducibility).

### Diurnal or nocturnal behaviour

A comprehensive phylogeny of Lepidoptera suggests that the ancestor of the monophyletic group of diurnal butterflies, nested within a paraphyletic group of (mostly) nocturnal moths, was likely nocturnal^49^. To evaluate differences in phenology between diurnal and nocturnal Lepidopterans, we first classified each species as either “butterfly” all of which were classified as diurnal, or “moth” most of which were classified a nocturnal, using the taxonomy of Aarvik et al.^71^. However, the moth dataset included 11 species that are primarily diurnal, according to Emmet^72^, and Hydén, et al.^73^. These species were *Adscita statices, Archiearis parthenias, Arctia plantaginis*, *Euclidia glyphica, E. mi, Hemaris fuciformis, Siona lineata, Zygaena exulans, Z. filipendulae, Z. lonicerae*, and *Z. viciae*, and they were all classified as diurnal together with the butterflies in the statistical analyses and comparisons.

### Voltinism

To evaluate and account for the effects of differences in voltinism on variation in phenology, we extracted information on the number of generations per year in different species from published sources^72,74–76^. In doing so, we accounted for the increasing incidence of bi/multivoltinism across northern Europe in recent decades. Species that produce one generation every two years were classified as semivoltine (0.5), species with one generation per year as univoltine (1), species that produce two generations or more per year as bivoltine (2), and species with a partial second generation were classified as bivoltine (2) as they may develop more than one generation per year. The percentage of species in our dataset that are semi-, uni- and multivoltine was comparable in diurnal (2, 78, and 20%) and nocturnal (0, 69, and 31%) Lepidopterans, and the incidence of obligately univoltine species was independent of diurnal group (Fisher’s exact, *P* = 0.13).

### Overwintering life stage

To evaluate and account for the effects of larval overwintering stage on variation in phenology, we extracted information on overwintering life stage (larva or not larva) of the different species from published sources^72–77^. The percentage of species in our dataset that overwinter as larva was higher among diurnal (64%, 51 of 80) than among nocturnal (35%, 98 of 283) species (χ^2^ = 21.86, df = 1, *P* < 0.0001). This was accounted for in the statistical analyses used to evaluate the effect of diel activity, outlined below.

### Statistical analysis and Reproducibility

Testing for differences in phenology according to taxonomic affinity. The species included in the present study represented 19 families (Drepanidae, Endromidae, Erebidae, Geometridae, Hesperiidae, Hepialidae, Lasiocampidae, Limacodidae, Lycaenidae, Noctuidae, Nolidae, Notodontidae, Nymphalidae, Papilionidae, Pieridae, Riodinidae, Saturniidae, Sphingidae, and Zygaenidae). To test whether the phenology of the adult flight period varies according to taxonomic affinity, general linear mixed model analysis of variance was implemented with procedure MIXED in SAS version 9.4^78–80^. Separate models were used for diurnal (*n* = 80) and nocturnal (*n* = 283) species, and for each phenology metric. The predictor variable was family (fixed effect). Species was included as a random (intercept) factor to account for non-independence of estimates for the beginning and end of the study period^20,78^. Approximate partial Eta-square local effect sizes (*η*^2^) were estimated sing procedure GLM in SAS^79,81^. A similar approach was used to estimate the signature of taxonomic affinity on long-term phenological shifts, based on the estimated intraspecific differences between 1981 and 2024, except that species was not included as a random factor in the model (only one estimate per species).

Evaluating pairwise associations between phenological variables. To investigate whether the different phenology metrics were independent, we performed pairwise Pearson correlation analyses. This was done separately for diurnal and nocturnal species. A correlation analysis was also used to investigate whether long-term shifts (number of days) in the onset were correlated or independent of corresponding shifts in the termination of the flight period.

Evaluating associations of phenology with year, diel activity, voltinism, and overwintering life stage. To evaluate long term shifts in phenology, separate paired *t*-tests were applied to each of the four phenology metrics, using data on the estimated intraspecific difference between 1981 and 2024. Separate analyses were used for diurnal and nocturnal species.

To evaluate whether the variation and change in the four phenology metrics were associated with year (1981 or 2024), diel activity, voltinism, and overwinter life stage, mixed model Anova’s (implemented with procedure MIXED in SAS) was applied to the predicted phenological metrics in 1981 and 2024 (i.e., using two estimates per species). All explanatory variables were included simultaneously. The interaction between year and diel activity was included to specifically evaluate whether long-term shifts were different in diurnal and nocturnal species. Results from mixed model Anova’s indicated that taxonomic affinity (family) accounted for 16% and 4.8%, on average, of the total variance in long term phenological shifts in diurnal and nocturnal species (Table S1). This resembles the results of a recent study of phenological shifts of 289 species of Finnish butterflies and moths^28^. These authors applied phylogenetic generalized least squares PGLS^82^; models and report that the signal for phylogenetic relatedness for phenological shifts was close to zero and far from significant (λ = 0.012, *P* = 0.78)^28^. Phylogenetic autocorrelation was also low in a study of phenology of moths and butterflies across the eastern USA^36^. To partially account for similarity according to shared relatedness, we nevertheless included family as a random effect in the model^20,83^. Species was included as an additional random (intercept) effect to account for non-independence of estimates for the beginning and end of the study period. The Satterthwaite method was used to approximate the denominator degrees of freedom for fixed effects. *P*-values were adjusted for multiple comparisons using Bonferroni corrections. The Wald test was used to assess the statistical significance of the random effects of family and species in each model^78^ to allow for comparisons with other studies. Approximations of local effect sizes for the fixed explanatory variables were estimated with the partial Eta-square statistic (Cohen’s *η*2) using procedure GLM in SAS^79,81^. Each phenology metric was analysed separately.

Evaluating intraspecific associations of contemporary phenology with latitude. To analyse intraspecific associations of contemporary phenology with latitude, we used the subset of diurnal (*n* = 54) and nocturnal (*n* = 176) species for which estimates of the four phenology metrics in 2024 could be predicted for all three latitudinal bands (55, 60 and 65 °N). First, the procedure MIXED in SAS was applied to evaluate whether and how phenology varied according to latitude, diel activity, and the interaction between latitude and diel activity. In this approach, the interaction between latitude and diel activity answers the question of whether latitudinal shifts are different in diurnal and nocturnal species. Species was included as a random factor (intercept) to account for repeated measures (three latitudes). Next, to evaluate whether results were robust to the inclusion of variation in phenology associated with other species traits and taxonomic affinity, more complex mixed models were fitted. In addition to latitude, diel activity, and their interaction, these models included voltinism and overwinter life stage (larva or not larva) as explanatory variables, and species and family were included as random effects. The onset, peak, termination, and duration of the flight period were analysed separately. *P*-values were adjusted for multiple comparisons using Bonferroni corrections.

Graphs and visualization of results were created using PowerPoint and SigmaPlot 15.0.

The data, R code, and SAS code used for the statistical analysis are available at (10.6084/m9.figshare.26097532) ^63^.

### Reporting summary

Further information on research design is available in the Nature Portfolio Reporting Summary linked to this article.

## Supplementary information

Supplementary Information

Description of Additional Supplementary Files

Supplementary Data 1

Reporting Summary

## Data Availability

The data, R code, and SAS code used for the statistical analysis supporting is publicly available at Figshare (10.6084/m9.figshare.26097532)^63^. The source data used to create the graphs in the main text is available as a supplementary data file (Supplementary Data 1).

## Code availability

The R and SAS code is publicly available at Figshare (10.6084/m9.figshare.26097532)^63^.
