# Supplementary material for: Contrasting phenological shifts in diurnal and nocturnal Lepidoptera under climate change
Source: Commun Biol. 2026 Apr 16;9:538. doi: 10.1038/s42003-026-10062-w (PMC13087129; doi:10.1038/s42003-026-10062-w)
Supplement: Supplementary file 1 — Supplementary Information [file 42003_2026_10062_MOESM1_ESM.pdf]

Supporting Information for

Contrasting phenological shifts in diurnal and nocturnal Lepidoptera under climate change

Anders Forsman[a]*, Bafraw Karimi[a], Markus Franzén[a,b]

[a] Center for Ecology and Evolution in Microbial Model Systems, EEMiS, Department of Biology and Environmental Science, Linnaeus University, SE-391 82 Kalmar, Sweden,
[b] Linköping University, IFM Biology, Linköping, Sweden,
***Correspondence**: Anders.Forsman@lnu.se

Table of contents:

**Table S1.** Associations of the four different phenology metrics (onset, termination, peak, and duration of the flight period) with taxonomic affinity (family).	Page 2
**Table S2.** Long-term phenological shifts of the adult flight period in diurnal and nocturnal Lepidopterans according to paired *t*-tests.	Page 3
**Table S3.** Pairwise correlations between the four phenology metrics in diurnal and nocturnal Lepidopterans.	Page 4
**Table S4.** Variation in phenological shifts in Lepidopterans according to diel activity, voltinism, and overwinter life stage.	Page 5
**Table S5.** Associations of contemporary phenology with latitude, diel activity, voltinism, and overwinter life stage.	Page 6
**Figure S1.** Trends in research output on phenology and climate change measured as publications per year up to December 2025.	Page 7
**Figure S2.** Variation in phenology according to family among the diurnal and nocturnal Lepidoptera species included in the study.	Page 8
**Figure S3.** Visualizations and comparisons of variation in the adult flight phenology of diurnal (n = 80) and nocturnal (n = 283) lepidopterans in 1980 and 2024.	Page 9
**Figure S4**. Relationship between variation in long-term shifts of the onset and termination of the flight period in diurnal and nocturnal species.	Page 10
**Supporting References**	Page 10

Table S1. Variation in phenology and phenological shifts according to taxonomic affinity (family) in diurnal (*n* = 80 species) and nocturnal (*n* = 283 species) Lepidopterans. Results from mixed model analyses of variance implemented with procedure MIXED in SAS. Phenology metrics include onset, termination, peak, and duration of the flight period. The predictor variables were family (fixed effect) and species (random effect, not included in the analyses of shifts). The table shows F-values, the Satterthwaite approximations for the denominator degrees of freedom (*df*), and *P*-values. P-values indicated in bold remained statistically significant after Bonferroni corrections (critical P = 0.05/16 = 0.0031). η^2 represent the approximate partial Eta-square local effect size estimated using procedure GLM in SAS [1,2]. The variation in phenology according to family is visualized separately for each group in **Fig. S2**.

**Effect of family**<br>Phenology attribute	*F*	*P*	η^2
Diurnal			
Onset	$F_{9,70} = 2.19$	0.0328	0.21
Termination	$F_{9,70} = 2.63$	0.0111	0.23
Peak	$F_{9,70} = 2.45$	0.0172	0.23
Duration	$F_{9,70} = 1.09$	0.3821	0.11
Shift Onset	$F_{9,70} = 2.72$	0.0089	0.26
Shift Termination	$F_{9,70} = 1.43$	0.1926	0.15
Shift Peak	$F_{9,70} = 0.51$	0.8622	0.06
Shift Duration	$F_{9,70} = 1.83$	0.0779	0.19
Nocturnal			
Onset	$F_{11,271} = 2.97$	**0.0010**	0.11
Termination	$F_{11,271} = 1.52$	0.1226	0.06
Peak	$F_{11,271} = 1.82$	0.0506	0.07
Duration	$F_{11,271} = 2.80$	**0.0018**	0.09
Shift Onset	$F_{11,271} = 2.22$	0.0139	0.08
Shift Termination	$F_{11,271} = 0.72$	0.7220	0.03
Shift Peak	$F_{11,271} = 1.17$	0.3097	0.05
Shift Duration	$F_{11,271} = 0.85$	0.5867	0.03

Table S2. Long-term phenological shifts in the flight activity period of diurnal (mainly butterflies) and nocturnal (moths only) Lepidopterans according to paired comparison *t*-tests. The phenological shifts are calculated as the difference (mean number of days (± s.d.)) between predicted phenology estimates (0.05, 0.5 and 0.95 percentiles) obtained for 2024 and 1981 obtained using separate quantile regressions for each species.

	Diurnal (n = 80)			Nocturnal (n = 283)		
Flight period	Change (s.d.)	*t*	*P*	Change (s.d.)	*t*	*P*
Onset	-0.23 (7.89)	-0.26	0.7969	2.10 (8.42)	4.20	<0.0001
Peak	-7.29 (7.47)	-8.73	<0.0001	2.31 (12.11)	3.20	0.0015
Termination	-7.89 (12.39)	-5.69	<0.0001	0.72 (14.31)	0.85	0.3975
Duration	-7.67 (15.11)	-4.54	<0.0001	-1.38 (16.08)	-1.45	0.1492

Table S3. Correlation matrices showing pairwise correlations between the four phenology metrics in diurnal (above the diagonal, 80 species x 2 year estimates = 160 observations) and nocturnal (below the diagonal, 283 species x 2 years = 566 observations) species. $p < 0.001$ (***), $p < 0.01$ (**), $p < 0.05$ (*).

	Onset	Termination	Peak	Duration
Onset		0.40<br>***	0.72<br>***	-0.48<br>***
Termination	0.83<br>***		0.85<br>***	0.62<br>***
Peak	0.92<br>***	0.93<br>***		0.20<br>*
Duration	-0.31<br>***	0.26<br>***	-0.005<br>0.91	

Table S4. Variation in phenological shifts in Lepidopterans according to diel activity, voltinism, and overwinter life stage. Results from mixed model analyses of variance implemented with procedure MIXED in SAS. Phenological shifts are calculated as the difference in ordinal date between quantile regression estimates (0.05, 0.5 and 0.95 percentiles) for 2024 and 1981. The predictor variables were diel activity (diurnal vs. nocturnal), voltinism, and overwinter life stage (larva vs. not larva). DF indicates the Satterthwaite approximations for the denominator degrees of freedom. All significant associations (highlighted in bold) remained statistically significant after Bonferroni corrections (critical *P* = 0.05/4 = 0.0125).

Phenological shift variable		Solution for Fixed Effects					
Onset	Diurnality	Overwinter	Estimate	S.E.	DF	t Value	Pr > |t|
Intercept			3.10	1.580	62.9	1.96	0.0544
Diel activity	diurnal		-1.09	1.424	12.4	-0.76	0.4596
Diel activity	nocturna		0	.	.	.	.
Overwinter		larva	**-3.66**	**0.922**	**320**	**-3.97**	**<0.0001**
Overwinter		not_larva	0	.	.	.	.
Voltinism			0.54	0.953	356	0.56	0.5746
Peak	Diurnality	Overwinter	Estimate	S.E.	DF	t Value	Pr > |t|
Intercept			-4.33	1.928	359	-2.25	0.0252
Diel activity	diurnal		**-8.72**	**1.444**	**359**	**-6.04**	**<0.0001**
Diel activity	nocturna		0	.	.	.	.
Overwinter		larva	-0.75	1.228	359	-0.61	0.5431
Overwinter		not_larva	0	.	.	.	.
Voltinism			**5.25**	**1.291**	**359**	**4.07**	**<0.0001**
Termination	Diurnality	Overwinter	Estimate	S.E.	DF	t Value	Pr > |t|
Intercept			-10.89	2.352	359	-4.63	<0.0001
Diel activity	diurnal		**-8.31**	**1.761**	**359**	**-4.72**	**<0.0001**
Diel activity	nocturna		0	.	.	.	.
Overwinter		larva	2.51	1.498	359	1.68	0.0942
Overwinter		not_larva	0	.	.	.	.
Voltinism			**8.17**	**1.575**	**359**	**5.18**	**<0.0001**
Duration	Diurnality	Overwinter	Estimate	S.E.	DF	t Value	Pr > |t|
Intercept			-13.44	2.700	131	-4.98	<0.0001
Diel activity	diurnal		**-7.14**	**2.036**	**26.7**	**-3.51**	**0.0016**
Diel activity	nocturna		0	.	.	.	.
Overwinter		larva	**6.26**	**1.714**	**330**	**3.65**	**0.0003**
Overwinter		not_larva	0	.	.	.	.
Voltinism			**7.49**	**1.801**	**357**	**4.16**	**<0.0001**

Table S5. **Associations of contemporary phenology with latitude, diel activity, voltinism, and overwinter life stage (larva or not larva).** Results from PROC MIXED in SAS based on data for 54 species of diurnal (mainly butterflies) and 176 species of nocturnal (moths only), all of which contributed with phenology estimates for three latitudinal bands (centred at 55, 60 and 65°N) in 2024. The analysed phenology attributes were start, end, peak, and duration of the flight period, estimated using quantile regression. The predictor variables were latitude (˚N), diel activity (diurnal / nocturnal), voltinism (0.5, 1 or 2), and overwintering stage (larva / not larva). The Satterthwaite approximation was used for the denominator degrees of freedom. *P*-values less than 0.05 are highlighted in bold. The random effect of species was significant for all phenological measures (all *P* < 0.0001).

Phenology variable	Type 3 Tests of Fixed Effects			
Effect	Num DF	Den DF	F Value	Pr > F
Onset				
Latitude	1	455	**139.49**	**<0.0001**
Diel activity (DA)	1	622	**6.66**	**0.0101**
Voltinism	1	228	**23.43**	**<0.0001**
Overwinter	1	273	1.55	0.2136
Latitude*DA	1	455	0.34	0.5580
Termination	Num DF	Den DF	F Value	Pr > F
Latitude	1	445	**8.14**	**0.0045**
Diel activity (DA)	1	300	**7.49**	**0.0066**
Voltinism	1	210	**5.12**	**0.0246**
Overwinter	1	206	2.37	0.1256
Latitude*DA	1	445	1.54	0.2149
Peak	Num DF	Den DF	F Value	Pr > F
Latitude	1	456	**6.28**	**0.0126**
Diel activity (DA)	1	585	**13.77**	**0.0002**
Voltinism	1	228	0.49	0.4826
Overwinter	1	265	1.74	0.1878
Latitude*DA	1	456	**5.42**	**0.0203**
Duration	Num DF	Den DF	F Value	Pr > F
Latitude	1	453	**120.14**	**<0.0001**
Diel activity	1	436	0.53	0.4651
Voltinism	1	216	**157.14**	**<0.0001**
Overwinter	1	221	0.05	0.8159
Latitude*DA	1	453	0.50	0.4817

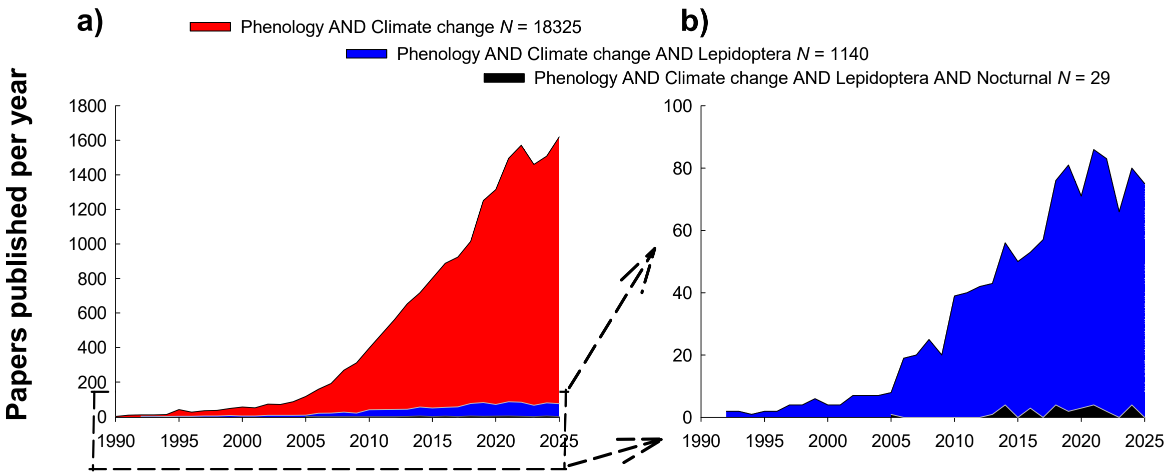

Figure S1. Trends in research output on phenology and climate change measured as publications per year up to December 2025. a) Absolute annual research output for all studies regardless of taxa (red) and for studies of Lepidoptera (blue). b) Absolute annual research output for studies of Lepidoptera (blue) and for studies of Lepidoptera addressing some aspect of nocturnal or crepuscular behaviour (black). Note that the scaling of the vertical axis is different in the two panels. Data extracted from a topic search conducted 16 March 2026 in ISI Web of Science (Data base: All; Time span: 1900-2025) using the following search strings:

1. Phenolog* AND (“Climate change” OR “Global warming”), generated 18325 papers (red).
2. Phenolog* AND (“Climate change” OR “Global warming”) AND (Lepidoptera* OR Butterfl* OR moth*), generated 1140 papers (blue).
3. Phenolog* AND (“Climate change” OR “Global warming”) AND (Lepidoptera* OR Butterfl* OR moth*) AND (nocturnal OR crepuscular OR night), generated 29 papers (black).
4. Phenolog* AND (“Climate change” OR “Global warming”) AND (nocturnal OR crepuscular OR night), generated 382 papers (not shown).

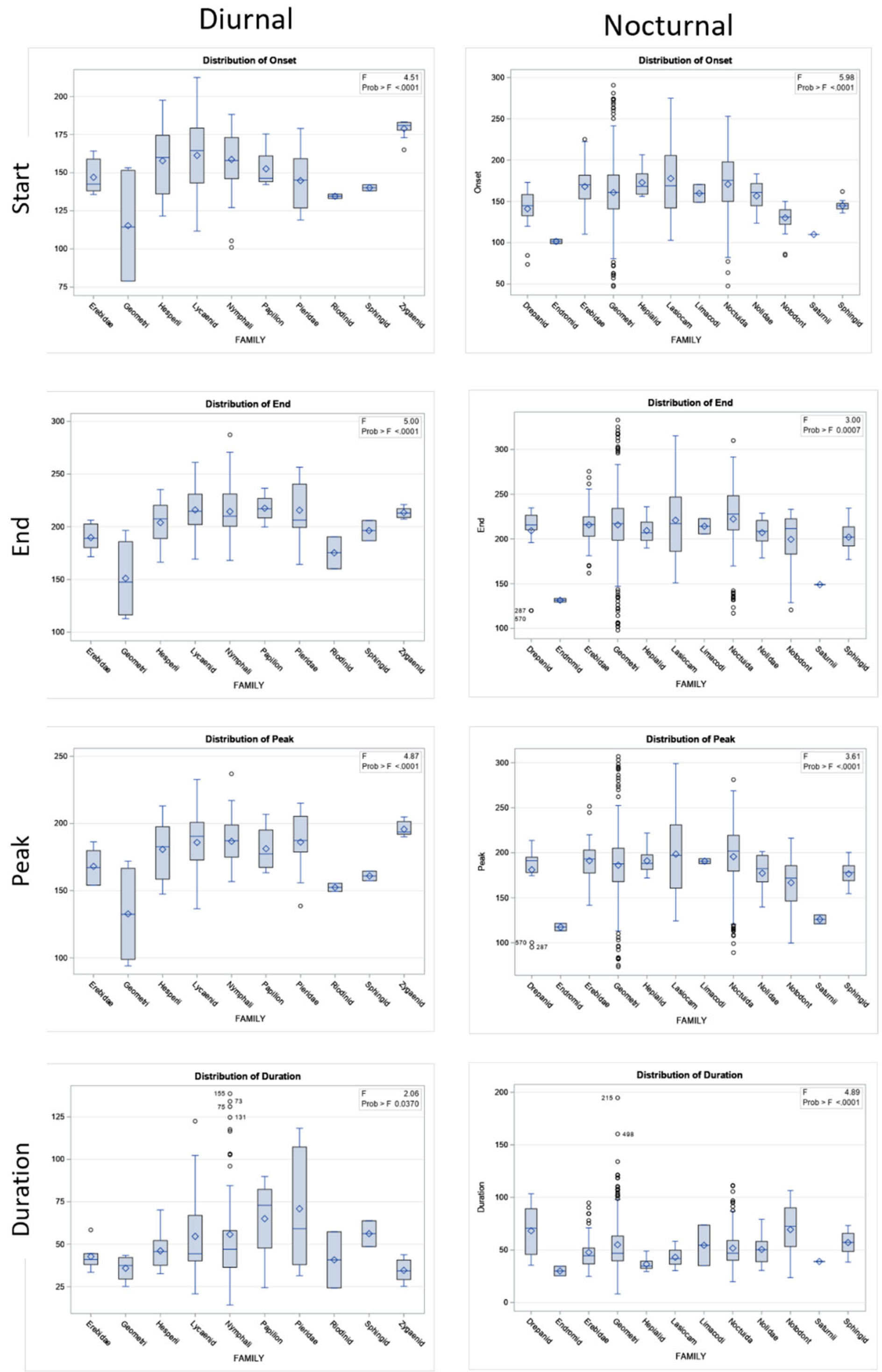

Figure S2. Variation in phenology according to family among the diurnal and nocturnal Lepidoptera species included in this study. The figure shows box plots by family for the onset (start), termination (end), peak, and duration of the adult flight period estimated at the beginning (1981) and end (2024) of the study. The onset (start), peak, and termination (end) were estimated using quantile regressions as the 0.05, 0.5 and 0.95 percentiles, in ordinal days. The duration was estimated by subtracting the onset from the termination of the flight period. Statistical results are reported in **Table S1**. Plots show centre line, median; diamond, mean; box limits, upper and lower quartiles; whiskers, 1.5x interquartile range; points, outliers.

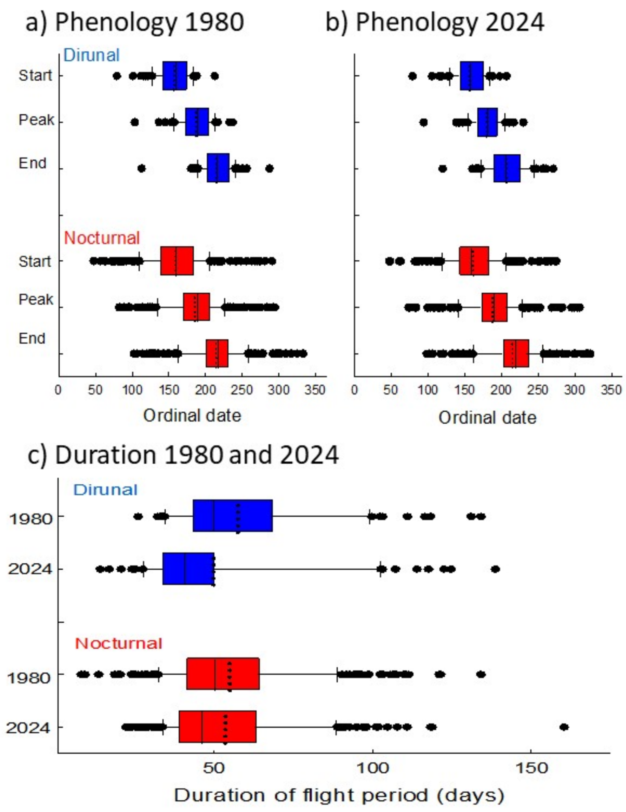

Figure S3. Visualizations and comparisons of variation in the adult flight phenology of diurnal (*n* = 80) and nocturnal (*n* = 283) Lepidopterans in Sweden in 1981 and 2024. The figure shows box plots for the predicted onset (start), peak, and termination (end) of the flight period in **a)** 1981 and **b)** 2024, estimated as the 0.05, 0.5 and 0.95 percentiles obtained using separate quantile regressions for each species. **c)** The duration of the flight period was estimated by subtracting the onset from the termination. Plots show centre line, median; dotted line, mean; box limits, upper and lower quartiles; whiskers, 1.5x interquartile range; points, outliers.

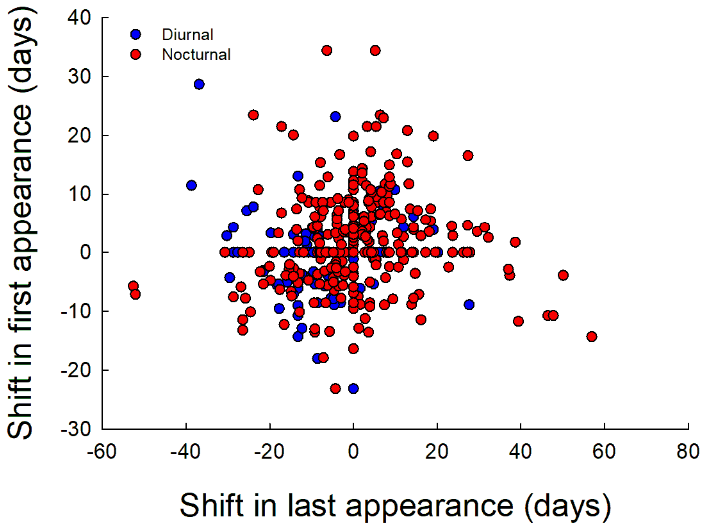

Figure S4. Variation in long-term shifts (between 1981 and 2024) of the estimated onset (first appearance) and termination (last appearance) of the flight period in diurnal and nocturnal Lepidopterans. There was no statistically significant relationship between temporal modifications of the onset (shift in first appearance) and the termination (shift in last appearance) of the flight period (Pearson correlation, Diurnal: r = -0.063, P = 0.58, n = 80; Nocturnal: r = -0.07, P = 0.24, n = 283).

Supporting References

1 Cohen, J. E. *Statistical power analysis for behavioral sciences*. (Lawrence Erlbaum Associates, Inc., 1988).

2 SAS_Institute_Inc. SAS/STAT 15.3 User's Guide. (SAS Institute Inc. North Carolina, 2023).
