# Supplementary material for: Contrasting phenological shifts in diurnal and nocturnal Lepidoptera under climate change
Source: Commun Biol. 2026 Apr 16;9:538. doi: 10.1038/s42003-026-10062-w (PMC13087129; doi:10.1038/s42003-026-10062-w)
Supplement: Supplementary file 4 — Reporting Summary [file 42003_2026_10062_MOESM4_ESM.pdf]

nature portfolio

Corresponding author(s): COMMSBIO-25-0989B

Last updated by author(s): Mar 31, 2026

Reporting Summary

Nature Portfolio wishes to improve the reproducibility of the work that we publish. This form provides structure for consistency and transparency in reporting. For further information on Nature Portfolio policies, see our Editorial Policies and the Editorial Policy Checklist.

Statistics

For all statistical analyses, confirm that the following items are present in the figure legend, table legend, main text, or Methods section.

n/a	Confirmed	
☐	☒	The exact sample size (*n*) for each experimental group/condition, given as a discrete number and unit of measurement
☒	☐	A statement on whether measurements were taken from distinct samples or whether the same sample was measured repeatedly
☐	☒	The statistical test(s) used AND whether they are one- or two-sided<br>*Only common tests should be described solely by name; describe more complex techniques in the Methods section.*
☐	☒	A description of all covariates tested
☐	☒	A description of any assumptions or corrections, such as tests of normality and adjustment for multiple comparisons
☐	☒	A full description of the statistical parameters including central tendency (e.g. means) or other basic estimates (e.g. regression coefficient) AND variation (e.g. standard deviation) or associated estimates of uncertainty (e.g. confidence intervals)
☐	☒	For null hypothesis testing, the test statistic (e.g. *F*, *t*, *r*) with confidence intervals, effect sizes, degrees of freedom and *P* value noted<br>*Give P values as exact values whenever suitable.*
☒	☐	For Bayesian analysis, information on the choice of priors and Markov chain Monte Carlo settings
☒	☐	For hierarchical and complex designs, identification of the appropriate level for tests and full reporting of outcomes
☐	☒	Estimates of effect sizes (e.g. Cohen's *d*, Pearson's *r*), indicating how they were calculated

Our web collection on statistics for biologists contains articles on many of the points above.

Software and code

Policy information about availability of computer code

Data collection	Phenological observations for Lepidoptera were assembled spanning ca 14 degrees latitude (55° to 69°) across Sweden, northern Europe, covering the period January 1981 to December 2024. For the period 1981–2020, we relied on our own curated historical dataset originally compiled via Analysportalen (https://fynddata.artdatabanken.se/analysisportal, now discontinued). On 10th October 2022, we extracted 2 298 332 records (covering 1 088 species, for the period from 1st January 1981 to 31st December 2020) which we carefully harmonized and quality-checked to correct for taxonomic inconsistencies and observational biases. For the period 2021–2024, we used records extracted from the Global Biodiversity Information Facility (GBIF, https://www.gbif.org/, DOI: 10.15468/dl.pfc2ve; extracted 7 June 2025), which by then had undergone substantial improvements in data quality and taxonomic validation. The combined dataset generated by this this two-tier approach ensured reliable long-term coverage while minimizing the risk of artefacts, since earlier records and online submissions often required extensive curation, whereas post-2020 records can be considered of consistently higher quality. We focused on conspicuous macro-Lepidoptera species that are reliably recorded in Swedish monitoring programmes and citizen science initiatives.<br>After applying taxonomic harmonisation and quality filtering (see Methods in Manuscript), the combined dataset comprised 363 species and 1,785,185 records spanning 1981–2024. This included 80 diurnal species (69 butterflies and 11 diurnal macro-moths) and 283 nocturnal macro-moth species representing 19 families.<br><br>The data, R code, and SAS code used for the statistical analysis supporting is publicly available at Figshare https://doi.org/10.6084/m9.figshare.26097532.
Data analysis	Quantile regression analysis implemented with R software was applied to estimate four phenology metrics (onset, peak, termination, and duration of the adult flight period) corresponding to the 0.05, 0.5, 0.95 and the difference between the 0.95 and the 0.05 quantiles) for each species at two time points (1981 and 2024) and separately for each species at three different latitudinal bands (in 2024). The generated

estimates were used in downstream analysis of long-term phenology shifts and contemporary intraspecific latitudinal trends, performed using SAS version 9.4.
All data, R code, and SAS code used for the statistical analysis supporting the results is publicly available at Figshare https://doi.org/10.6084/m9.figshare.26097532

For manuscripts utilizing custom algorithms or software that are central to the research but not yet described in published literature, software must be made available to editors and reviewers. We strongly encourage code deposition in a community repository (e.g. GitHub). See the Nature Portfolio guidelines for submitting code & software for further information.

Data

Policy information about availability of data

All manuscripts must include a data availability statement. This statement should provide the following information, where applicable:

- Accession codes, unique identifiers, or web links for publicly available datasets
- A description of any restrictions on data availability
- For clinical datasets or third party data, please ensure that the statement adheres to our policy

The data, R code, and SAS code used for the statistical analysis supporting the results The data, R code, and SAS code used for the statistical analysis supporting is publicly available at Figshare https://doi.org/10.6084/m9.figshare.26097532

Research involving human participants, their data, or biological material

Policy information about studies with human participants or human data. See also policy information about sex, gender (identity/presentation), and sexual orientation and race, ethnicity and racism.

Reporting on sex and gender	Not applicable - this study did not involve humans
Reporting on race, ethnicity, or other socially relevant groupings	Not applicable - this study did not involve humans
Population characteristics	Not applicable - this study did not involve humans
Recruitment	Not applicable - this study did not involve humans
Ethics oversight	Not applicable - this study did not involve humans

Note that full information on the approval of the study protocol must also be provided in the manuscript.

Field-specific reporting

Please select the one below that is the best fit for your research. If you are not sure, read the appropriate sections before making your selection.

☐ Life sciences ☐ Behavioural & social sciences ☒ Ecological, evolutionary & environmental sciences

For a reference copy of the document with all sections, see nature.com/documents/nr-reporting-summary-flat.pdf

Ecological, evolutionary & environmental sciences study design

All studies must disclose on these points even when the disclosure is negative.

Study description	We propose a model hypothesizing that dissimilar light requirements and constraints will differently impact phenological responses in diurnal versus nocturnal ectotherms. To evalaute model predictions, we next investigate temporal shifts and latitudinal variation in phenology for 363 Lepidoptera species using four decades of citizen science data.
Research sample	The combined dataset used included information on spatiotemporal variation in phenology of Lepidoptera and comprised 363 species and 1,785,185 records spanning 1981–2024, and 14 degrees latitude (55° to 69°) across Sweden. This sample used for analysis of long-term shifts included 80 diurnal species (69 butterflies and 11 diurnal macro-moths) and 283 nocturnal macro-moth species. The subsample used for analysis of contemporary intraspecific latitudinal phenology trends included records for 54 diurnal and 176 nocturnal species (n = 996,655 observations, from 2013 to 2024) that were assigned to one of three latitudinal bands (from decimal latitude: 52.5–57.5°N (55 °N band), 57.5–62.5°N (60 °N band), and 62.5–67.5 °N (65 °N band)), each spanning 5 °.
Sampling strategy	We relied on citizen science records of Lepidoptera. On 10th October 2022, we extracted 2 298 332 records from Analysportalen, covering 1 088 species of butterflies and moths, for the period from 1st January 1981 to 31st December 2020. On 7 June 2025, we extracted additional records covering the period 2021 to 2024 from the Global Biodiversity Information Facility (GBIF). To mitigate potential biases due to spatiotemporal variation in sampling intensity, quantile regression approaches were used to quantify the onset (0.05 percentile), peak (0.50 percentile), termination (0.95 percentile), and the duration (termination - onset) of the adult flight period.
Data collection	For phenology data covering the period 1981 to 2020 we relied on our own curated historical dataset originally compiled via the

Data collection	Swedish biodiversity database 'Analysportalen' (now discontinued, with parts of the resources and functionality moved to the Swedish Observation System, https://Artportalen.se, and the Swedish Biodiversity Data Infrastructure, https://biodiversitydata.se) , which we carefully harmonized and quality-checked to correct for taxonomic inconsistencies and observational biases. For the period 2021–2024, we used records extracted from the Global Biodiversity Information Facility (GBIF, https://www.gbif.org/, DOI: 10.15468/dl.pfc2ve; extracted 7 June 2025).<br>The data on species traits (diurnal/noctrunal, voltinism, overwintering strategy) were extracted for published sources, as specified in the manuscript.
Timing and spatial scale	Timing: the data used covered the period January 1981 to December 2024. Spatial scale: the data was collected for Sweden spanning 14 degrees latitude (55° to 69°), northern Europe.
Data exclusions	We filtered the observations as described under Data collection (for details see manuscript and the statistical code). None of the filtered data were excluded from the analyses.
Reproducibility	We describe the study, including data collection and statistical analyses, to make it easy to follow and to reproduce.
Randomization	Not applicable.
Blinding	Blinding was not possible because of the nature of the data used.

Did the study involve field work? ☐ Yes ☒ No

Reporting for specific materials, systems and methods

We require information from authors about some types of materials, experimental systems and methods used in many studies. Here, indicate whether each material, system or method listed is relevant to your study. If you are not sure if a list item applies to your research, read the appropriate section before selecting a response.

Materials & experimental systems

n/a	Involved in the study
☒	☐ Antibodies
☒	☐ Eukaryotic cell lines
☒	☐ Palaeontology and archaeology
☒	☐ Animals and other organisms
☒	☐ Clinical data
☒	☐ Dual use research of concern
☒	☐ Plants

Methods

n/a	Involved in the study
☒	☐ ChIP-seq
☒	☐ Flow cytometry
☒	☐ MRI-based neuroimaging

Plants

Seed stocks	Not applicable.
Novel plant genotypes	Not applicable.
Authentication	Not applicable.
